# Liquid Biopsy for Small Cell Lung Cancer either De Novo or Transformed: Systematic Review of Different Applications and Meta-Analysis

**DOI:** 10.3390/cancers13092265

**Published:** 2021-05-08

**Authors:** Elio Gregory Pizzutilo, Martino Pedrani, Alessio Amatu, Lorenzo Ruggieri, Calogero Lauricella, Silvio Marco Veronese, Diego Signorelli, Giulio Cerea, Laura Giannetta, Salvatore Siena, Andrea Sartore-Bianchi

**Affiliations:** 1 Niguarda Cancer Center, Grande Ospedale Metropolitano Niguarda, 20162 Milan, Italy; eliogregory.pizzutilo@ospedaleniguarda.it (E.G.P.); martino.pedrani@ospedaleniguarda.it (M.P.); alessio.amatu@ospedaleniguarda.it (A.A.); lorenzo.ruggieri@ospedaleniguarda.it (L.R.); calogero.lauricella@ospedaleniguarda.it (C.L.); silviomarco.veronese@ospedaleniguarda.it (S.M.V.); diego.signorelli@ospedaleniguarda.it (D.S.); Giulio.cerea@ospedaleniguarda.it (G.C.); lauragiuseppina.giannetta@ospedaleniguarda.it (L.G.); salvatore.siena@ospedaleniguarda.it (S.S.); 2 Department of Oncology and Hemato-Oncology, Università degli Studi di Milano, 20122 Milan, Italy

**Keywords:** circulating tumor cells, CTCs, circulating tumor DNA, ctDNA, histologic transformation, liquid biopsy, small cell lung cancer, transformed SCLC

## Abstract

**Simple Summary:**

Small cell lung cancer (SCLC) is an aggressive tumor, which can occur either de novo or from the histologic transformation of non-small cell lung cancer. Liquid biopsy has demonstrated its capability to detect, characterize and monitor different cancers. The aim of this systematic review was to assess the potential added value of liquid biopsy, in terms of circulating tumor DNA (ctDNA) and circulating tumor cells (CTCs), in the management of SCLC, either de novo or transformed. We found ctDNA analysis as the most valuable and feasible technology to be integrated into clinical for disease monitoring (response, relapse, transformation) or for genomic profiling of SCLC, with a potential use also for prognostic stratification. CTCs hold a strong prognostic significance, as confirmed by our meta-analysis (even if potentially biased), but the heterogeneity of available data, the lack of agreed cut-offs, and the less affordable technology make CTCs more difficult to be integrated into present clinical practice.

**Abstract:**

Background: The potential added value of liquid biopsy (LB) is not well determined in the case of small cell lung cancer (SCLC), an aggressive tumor that can occur either de novo or from the histologic transformation of non-small cell lung cancer (NSCLC). Methods: A systematic review of studies adopting LB in patients with SCLC have been performed to assess the clinical utility of circulating tumor DNA (ctDNA) or circulating tumor cells (CTCs). Results: After a screening of 728 records, 62 studies (32 evaluating CTCs, 27 ctDNA, and 3 both) met predetermined eligibility criteria. Only four studies evaluated LB in the diagnostic setting for SCLC, while its prognostic significance was evaluated in 38 studies and prominently supported by both ctDNA and CTCs. A meta-analysis of 11 studies as for CTCs enumeration showed an HR for overall survival of 2.63 (1.71–4.05), with a potential publication bias. The feasibility of tumor genomic profiling and the predictive role of LB in terms of response/resistance to chemotherapy was assessed in 11 and 24 studies, respectively, with greater consistency for those regarding ctDNA. Intriguingly, several case reports suggest that LB can indirectly capture the transition to SCLC in NSCLC treated with EGFR tyrosine kinase inhibitors. Conclusions: While dedicated trials are needed, LB holds potential clinical roles in both de novo and transformed SCLC. CtDNA analysis appears the most valuable and practicable tool for both disease monitoring and genomic profiling.

## 1. Background

Small cell lung cancer (SCLC) is an aggressive lung cancer with neuroendocrine features, characterized by a strong association with tobacco smoke exposure, high cell growth fraction, and early and extensive metastatic propensity. Despite the initial high rate of responsivity to cytotoxic chemotherapy and radiation therapy, the rapid development of resistance and the high rate of relapse justify the poor outcome overall [1]. Only 6% of 290,000 patients worldwide diagnosed annually with SCLC will survive 5 years from diagnosis, mostly those with the limited stage of disease (LS-SCLC) [2]. In addition, a subset of patients affected by oncogene-driven non-small-cell lung cancer (NSCLC) experiences a histologic transformation in SCLC, commonly under the therapeutic pressure of tyrosine kinase inhibitors (TKIs). Small-cell transformation represents an acquired mechanism of resistance to TKIs reported in 5–15% of *EGFR* mutated NSCLC. In these cases, transformed cells usually maintain the original activating *EGFR* mutation and present other genetic alterations resembling classical SCLC (e.g., mutations or loss of *RB1* and *TP53*, mutations of *PIK3CA*) [3,4]. While liquid biopsy (LB) is extensively used in patients with NSCLC to detect on-target resistance mechanisms, tissue re-biopsy appears crucial for identifying histologic transformations.

Given that nearly all SCLC patients are not treated with surgical resections, the shortage of banked tumor tissue has been an obstacle for comprehensive genomic characterization, hindering the understanding of the biology of SCLC and possibly slowing down preclinical drug development [5]. LB represents nowadays a minimally invasive tool for obtaining tumor-derived components such as circulating tumor DNA (ctDNA) or circulating tumor cells (CTCs) with different applications across many cancer types [6,7,8,9]. SCLC ctDNA can be identified and profiled by detecting either a single gene of interest (e.g., *TP53)* or a panel of SCLC-associated genes with technologies widely available in clinical laboratories [10,11]. The amount of ctDNA in a sample of circulating free DNA (cfDNA) can vary enormously between patients with lung cancer, reflecting the histologic type, the tumor burden, and the sites of disease [12,13]. CTCs are very rare in the bloodstream, but they can be isolated using marker-dependent approaches (e.g., by antibodies against epithelial proteins) or exploiting biophysical differences of CTCs from other blood cells [5]. The most adopted and the only FDA-approved system is the CellSearch platform, in which the epithelial cell adhesion molecule (EpCAM/CD326) is used for CTC detection [14]. CTCs derived from patients with SCLC maintain their tumorigenic properties in immunocompromised mice, also forming patient CTC-derived explants (CDX) [15], and several studies have demonstrated their enumeration as an independent prognostic biomarker for survival [16,17,18], albeit, a consensus on the optimal cut-off threshold is lacking. Other potential roles of ctDNA and CTCs are less clear, even though a number of studies have reported fragmented data on their diagnostic, genomic profiling, predictive, or prognostic value.

We performed a systematic review of the studies available in the literature evaluating LB as a clinical implement in the management of SCLC, including transformed SCLC from NSCLC.

## 2. Methods

### 2.1. Definition of the Outcome

The purpose is to evaluate the current potential added value of LB, in terms of circulating tumor DNA (ctDNA) and circulating tumor cells (CTCs), in patients with de novo SCLC or transformed SCLC.

### 2.2. Data Source and Search Strategy

A systematic review of the literature was performed according to PRISMA statement criteria [19,20] on 6 February 2021. The Medline and EMBASE databases were searched for relevant records between 2000 and 2021 that met the study inclusion/exclusion criteria. Unpublished data presented as abstracts in relevant international congresses (American Society of Clinical Oncology (ASCO), European Society of Medical Oncology (ESMO), and International Association for the Study of Lung Cancer (IASLC)) were included. Hand searches were performed to identify further potentially eligible studies, as well. The decision to include a study for review was made by consensus between two authors (EGP and MP). The research criteria were limited to human studies published only in the English language. The search terms were (“small cell” OR “small-cell” OR “SCLC” NOT “non-small”) OR (“transformed small cell” OR “transformed small-cell” OR “transformed SCLC” OR “histologic transformation”) AND (“ctDNA” OR “cfDNA” OR “circulating free DNA” OR “circulating tumor DNA” OR “CTC” OR “liquid biopsy”).

Main study inclusion criteria:

- Analysis of cfDNA/ctDNA or CTCs in plasma/serum of patients with SCLC included histologically transformed SCLC from NSCLC;
- Genomic profiling, diagnosis, treatment response, and/or survival data collected and correlated with cf/ctDNA or CTCs in humans.

Study exclusion criteria:

- Not specific for SCLC, except in the cases of small cell transformation;
- Analysis of tumoral circulating components different from ctDNA or CTCs.

For the quantitative analysis [21], we included in a meta-analysis of prognostic value those studies with comparable techniques used for ctDNA detection or CTC enrichment. The quality of studies was evaluated through the Nottingham–Ottawa Scale (NOS). The total scores ranged from 0 (worst) to 9 (best), with a score of at least seven indicative of high quality. Included studies reported:

- Hazard ratios (HR) with 95% confidence intervals (CI);
- Sample size;
- Cut-off of CTC number.

### 2.3. Statistical Analysis

Methods described by Tierney et al. [22] were adopted for the prognostic meta-analysis and for the collection of time-to-event data. All statistical analyses were performed using R software (R version 4.0.5) (R: A language and environment for statistical computing. R Foundation for Statistical Computing, Vienna, Austria. https://www.R-project.org/ accessed on 5 March 2021) with metafor package (Viechtbauer, W+010 https://www.jstatsoft.org/v36/i03/ accessed on 5 March 2021), dmetar package (Harrer, M., Cuijpers, P. et al. 2019 http://dmetar.protectlab.org accessed on 5 March 2021), and meta package (Schwarzer, G. 2020 https://CRAN.R-project.org/package=meta accessed on 5 March 2021). Tau-squared and I^2^ index were used to evaluate heterogeneity. Funnel plots with Egger’s regression tests were used to examine publication bias.

## 3. Results

**Figure 1:**
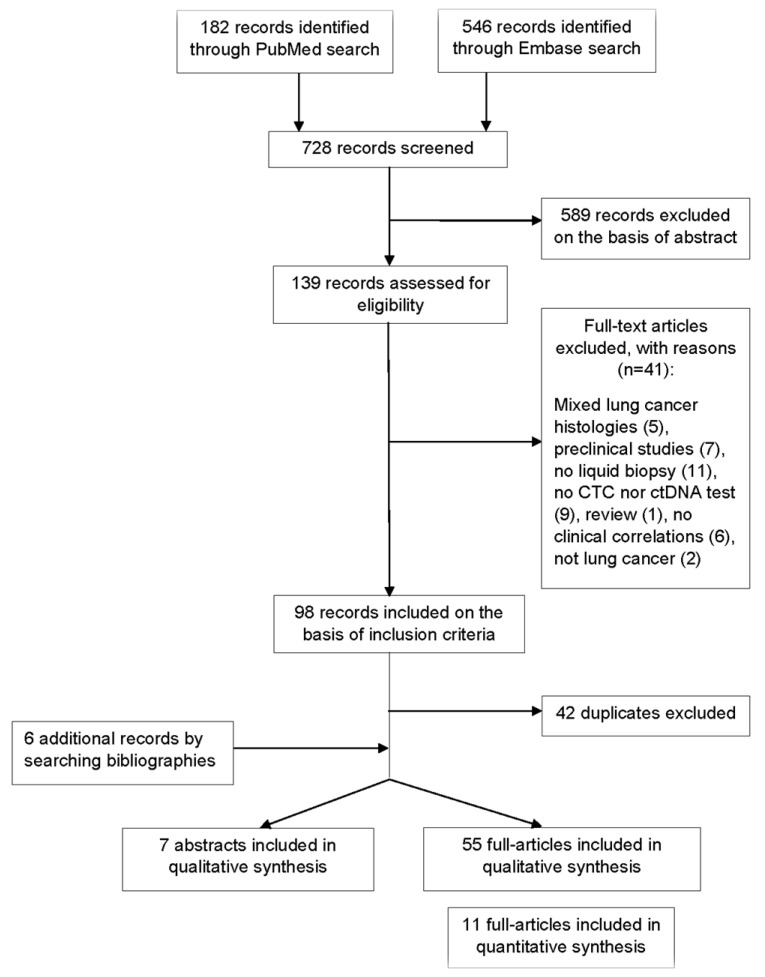
Flow diagram representing the systematic review process performed according to PRISMA statement.

A total of 728 records were screened to be included in the systematic review (Figure 1). According to selection criteria, we identified 56 records found through database searching (PUBMED and EMBASE) and 6 records by searching bibliographies. As a result, 62 records were eligible and included in the systematic review: 55 full-text articles studies and 7 abstracts presented at international congresses. Fifteen studies concerned transformed SCLC. Overall, identified records included mainly clinical trials with exploratory endpoints assessing the value of LB, observational studies, and case series or case reports for transformed SCLC. A total of 32 studies evaluated CTCs, 27 studies evaluated the role of circulating DNA, and 3 studies explored both (Tables S1 and S4).

The detection rate of ctDNA in patients harboring de novo SCLC varied between 49% and 100%, with a median of 91%. Among six studies in which next-generation sequencing (NGS) technology was adopted with panels of 5–430 genes, the detection rate was 71–100%, with a median of 91% (Table S2).

The detection rate of CTCs at baseline in 20 studies varied between 49% and 96%, with a median of 85%, with different assays. The median number of CTCs and the median detection rate with different assays, among studies with available data, in patients with extensive-stage SCLC (ES-SCLC) versus limited-stage SCLC (LS-SCLC), were 38 vs. 2, and 95% vs. 65%, respectively (Table S3).

We subdivided our results into five categories according to the investigated role of ctDNA or CTCs: diagnostic (studies including negative controls), genomic profiling, predictive of treatment response, and prognostic (in terms of disease recurrence or survival) for SCLC; the last category was related to the application of LB in cases of small-cell transformation of NSCLC.

### 3.1. Diagnostic

Four studies (Table S1) compared the detectability of ctDNA or CTCs from patients with SCLC and from healthy volunteers. With overall 172 SCLC patients and 176 non-cancer controls, these works described in the plasma of patients the presence of a higher DNA concentration (with longer fragments), more frequent *TP53* mutations (36% of patients with early-stage SCLCand 11% of non-cancer controls), detectable tumor-related mutations and CNAs by NGS in 84–94% of cases versus 0 in non-cancer controls, and detectable CTCs in 86% of cases versus 0% in healthy donors [10,11,23,24].

### 3.2. Genomic Profiling

Overall, 11 studies reported data of genomic profiling of SCLC by LB (Table S1). Different authors presented results of ctDNA analysis by NGS [10,12,25,26,27,28,29]. A higher concordance (median of 94% of paired mutations) between ctDNA and tissue was reported to be reachable by means of a deep sequencing [28,30], also showing a correlation in allelic frequency (AF) of gene mutations between plasma and tissue [28]. Some mutations were exclusively detected in ctDNA, and, comparing samples collected at baseline and after treatment, new mutations appeared or became dominant in the post-treatment samples [28,30]. Mohan and colleagues identified potential therapeutic targets in >50% of patients [10]. Devarakonda et al. provided the widest study of genomic profiling of SCLC using Guardant360 platform on ctDNA from 564 patients and highlighted the identification of potentially targetable alterations involving the androgen receptor gene (*AR*), the RTK/RAS pathway, or genes involved in DNA repair [26]. The most frequent genetic alterations detected by means of ctDNA analysis in seven studies through different assays [10,12,25,26,27,28,29] are reported in Table 1 and Table 2.

An association between CTC count and quantity of circulating free DNA (cfDNA) has been described in a cohort of 12 patients; moreover, identical genetic alterations were identifiable from both CTC-derived DNA and cfDNA [31]. Su et al. demonstrated the feasibility of genomic profiling by means of single-cell sequencing of CTCs in SCLC patients, with 68–99% of mutations observed in tissues detectable in CTCs. The authors observed that CTCs mainly disseminated from the primary tumor with which they shared the majority of the mutations, while metastatic sites formed minor clones unobserved in CTCs [32].

### 3.3. Predictive

We found 24 studies reporting data about the predictive potential of LB (Table S1). Several investigators reported a correlation between qualitative and quantitative changes in ctDNA and variation in tumor burden under treatments [10,12,28,29,33,34]. Hence, a rise in detection rate and also in AFs or copy number of DNA alterations may precede clinical evidence of disease progression; on the other hand, a reduction can be observed in cases of response to chemotherapies [10,12,28]. Herbreteau et al. reported a significantly lower activity of atezolizumab in those patients with detectable ctDNA in II line setting, while no differences existed with chemotherapy [29]. Thomas et al. described a case of *BRCA1*-mutated SCLC treated within a phase II trial with olaparib and durvalumab. The patient achieved a complete response with a decline in the frequency of *BRCA1* mutation in ctDNA [34]. One group found distinct qualitative features by ctDNA analysis in patients with chemosensitive or chemorefractory SCLC. Alterations of *TP53*, *ATM*, and *FLCN* were higher in the chemorefractory group and related with shorter PFS, while *APC* abnormalities occurred more frequently among chemosensitive patients [35]. Owonikoko et al., in a negative randomized phase II trial with paclitaxel in addiction either to alisertib (inhibitor of Aurora A kinase, a regulator of mitosis) or placebo in relapsed SCLC, retrospectively reported better survival in patients with alterations of the genes involved in cell cycle regulation among patients treated with alisertib [25].

A lack of significance in the predictive role of the CTC number assessed by CellSearch at baseline was described in four out of five studies [17,36,37,38,39]. Most of the studies exploring the significance of CTC count modification using CellSearch, reported a reduction in enumeration after chemotherapy [17,18,40,41], but no statistically significant differences among groups showing different responses to treatment [17,18,37,38,39,42]. A correlation between tumor burden in response to therapy and CTC number by CellSearch was observed only in II-III line setting among patients treated, respectively, with temozolomide +/− veliparib and with pazopanib [43,44,45]. Conversely, adopting assays different from CellSearch, a significant correlation with disease response and changes in CTC count can be found. Incremental CTC count has been reported using a method based on the specific telomerase activation of cancer cells (OBP-401) [46], while folate receptor-positive CTCs [47], CK-19 mRNA-positive CTC [48], or DLL3+/CD45− CTCs [49], decrease during response. Finally, different profiles of copy number aberrations (CNAs) in CTCs and in ctDNA have been associated with different responsiveness to first-line chemotherapy by three groups [32,35,50].

### 3.4. Prognostic

This was the most explored purpose of LB with overall 38 clinical reports available (9 by ctDNA, 27 by CTC, 2 by both) (Table S1). Two studies, with overall 49 patients, reported a significant association between a higher abundance of cfDNA/ctDNA at baseline and shorter OS and PFS [12,28]. Palma presented data of survival benefit among patients with a continuous drop in ctDNA levels during chemotherapy [51]. Contrasting prognostic connotations have been reported for total mutation burden, mutations of specific genes, or AFs of certain mutations, while the CNA signature measured by ctDNA sequencing could predict survival [10,25,28,33,52,53]. Alterations in *SETBP1*, *PBRM1*, *ATRX*, *EP300*, *ATM, PIK3CA/G*, or *NOTCH1* have been associated with OS or PFS in patients treated with platinum-based chemotherapy or with chemoradiation by two small studies [27,52]. A French group observed a worse OS among patients with relapsed SCLC and detectable mutations through an NGS panel limited to *NOTCH1-3*, *TP53*, and *RB1* genes [29]. After the first description of prognostic significance hold by CTCs detected by flow cytometry [54], several groups reported a significant correlation between survival and the number of CTCs found by CellSearch, regardless of the statistical method applied. Baseline pretreatment CTC number consistently correlated with OS in 15 studies (including ES and/or LS-SCLC) with different cut-offs (from 2 CTCs up to 282 CTCs) [10,16,17,36,37,38,40,42,55,56,57,58,59,60,61]. A higher number of CTCs after the first cycle of chemotherapy was also prognostic for worse outcomes in six studies [16,18,42,57,58,60]. Overall, six studies reported a prognostic significance of CTCs enumeration post-second cycle, post-treatment, and at the time of relapse [17,18,36,37,59,61]. Some authors observed a prognostic significance not only in the absolute number of CTCs but also in its reduction during chemotherapy [16,17,60]. Gadgeel et al., evaluating pembrolizumab as maintenance treatment after platinum and etoposide, collected CTCs before the first three cycles of pembrolizumab. No correlation with PFS and OS was found with any CTC count before or during immunotherapy [62]. Two groups described a strong prognostic value of CTCs in LS-SCLC treated with CRT [55,56]. In the second-line setting, Aggarwal presented data showing a lack of association between CTC count and survival in a small cohort of patients with relapsed/refractory SCLC [31]. Two groups, evaluating the activity of second-line pazopanib and second-line temozolomide in combination with veliparib or with placebo, respectively, reported a correlation between a number of CTCs ≥5 both before treatment and after the first cycle with a significantly shorter OS [43,45]. Different groups explored the prognostic value of distinct phenotypes of CTCs. Detectable BCL2+ or DLL3+ or chromosome 8 centromere probe (CEP8)+ and CD45- CTCs correlated with worse outcome at baseline and/or after first cycle of first-line chemotherapy [49,57,63]. No correlation was found with TTF-1+, or CD56+, or pancytokeratin+, and CD45- CTCs [23,36]. Igawa and colleagues observed a prolonged OS in patients with <2 CTCs at baseline, using the aforementioned OBP-401 assay [46]. Shen et al., by means of an LT-PCR to detect folate receptor-positive CTCs, found a trend for longer OS (*p* = 0.056) in patients with low CTC level at baseline, while no differences were evident between patients with positive or negative CTC count [47]. Shi et al. described a strong prognostic value of CK19 mRNA-positive CTCs (HR for OS 3.31 when detectable after treatment) [48]. One group also reported a correlation between the presence of CK+/Vim+ CTCs after one cycle of second-line pazopanib and shorter survival [44]. In addition, copy number aberrations (CNA) signatures measured in CTCs from pretreatment blood samples could predict PFS and OS [32,50].

#### Meta-Analysis

**Figure 2:**
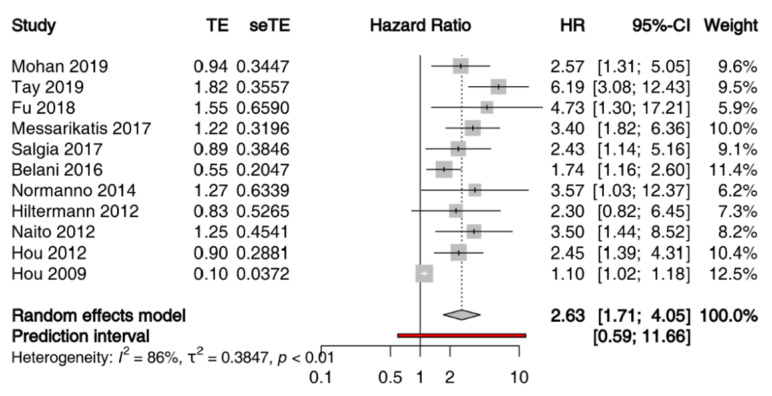
Prognostic significance of CTCs assessed by CellSearch in patients with SCLC at baseline: forest plot of hazard ratios for overall survival.

Given the heterogeneity of techniques adopted for quantitatively and qualitatively evaluation of ctDNA and of time points of sample collection, a meta-analysis of the prognostic value of ctDNA in patients with SCLC was not feasible. Conversely, 11 studies comprising 861 patients met the eligibility criteria for a meta-analysis of the prognostic value of CTCs assessed by CellSearch (Table S4). We performed a meta-analysis of HR for OS of the number of CTCs at baseline, with different cut-offs across studies. When multiple cut-offs were explored in the same study, we chose those with the better operating characteristics throughout ROC analysis for the meta-analysis. All studies included patients with LS-SCLC, or ES-SCLC treated with chemoradiation or first-line platinum etoposide (+/− experimental drug if it did not significantly affected survival) [38,42]. The sample size per study ranged from 51 to 120 patients, and studies were published between 2009 and 2019. Because the heterogeneity across the studies was high (I^2^ = 86%, *p* < 0.01), the estimated pooled HR was calculated using a random-effect model. The pooled HR showed that the presence of CTCs correlated with reduced OS (HR = 2.63; 95% CI: 1.71–4.05) (Figure 2). Moreover, the visual inspection of the funnel plot and the Egger test demonstrated the presence of a potential publication bias (Figure S1).

### 3.5. Small-Cell Transformation of NSCLC

Our group and others described cases of patients with progressive NSCLC under TKI treatment with evidence of sharp elevation of AF of *EGFR* activating mutation in ctDNA measured with ddPCR, concurrent with evidence of histologic transformation to SCLC [64,65,66]. Performing NGS on ctDNA, other researchers also reported a significant elevation in *EGFR* mutation AF concomitant with the increase in AF of *TP53*, *RB1*, or *PIK3CA* alterations before or at the moment of the histologic transformation, after TKI failure [67,68,69,70,71]. A subsequent reduction in the allelic abundance of these mutations after an SCLC-directed treatment has been reported, as well [69,70]. Two groups presented data supporting the utility of ctDNA in detecting the *EGFR* resistant mutation T790M even after SCLC transformation, uncovering the spatial heterogeneity of the tumor [72,73], while one group described the disappearance of acquired G1202R *ALK* mutation, concurrent with SCLC transformation [74]. We also found two studies demonstrating that CTC phenotyping and single-cell CTC sequencing could suggest a histologic transformation. Ni et al., through a single-cell exome sequencing, identified *EGFR*, *PIK3CA*, *RB1,* and *TP53* mutations in CTCs of a patient with transformed SCLC, with higher abundance than in the original NSCLC [75]. More recently, a Chinese group applied a new assay allowing rapid CTC isolation with different aptamers and characterization by immunocytochemistry. They firstly reported a significant reduction in mean nuclear size and a phenotype shift of CTCs concurrent with SCLC transformation in 14 patients [76] (Table 3).

## 4. Discussion

To our knowledge, this is the first systematic review concerning the applications of LB (considering both ctDNA and CTCs) focused on SCLC, also innovatively including patients affected by transformed SCLC. The limitations of data retrieved are mostly related to the variety of techniques adopted for LB, diversity of patient populations (ES or LS-SCLC, different lines of treatment) or limited sample size, the timing of plasma samples (e.g., at baseline, during, or after treatments) and heterogeneous approaches to statistical analyses. With such considerations, we found interesting potential applications for each purpose of the five explored.

### 4.1. Diagnosis

Among the potential applications of LB, cancer diagnosis and screening represent the greatest challenge in oncology. Even though SCLC presents relatively higher concentrations of tumor-derived components in plasma, the presence of mutations in cfDNA potentially associated with non-malignant processes and the elevated growth rate with an early metastatic propensity of this cancer type add obstacles to the feasibility of a screening program. Limited data support this application for LB in SCLC to date. Mohan and colleagues recently demonstrated high sensitivity and specificity with multi-gene NGS of ctDNA, in contrast with previous data on single gene sequencing [11], describing detectable tumor-related CNAs in 84% and non-synonymous mutations in 94% of patients (77% and 91% in LS-SCLC), versus 0% in non-cancer controls [10]. No other studies have focused on the diagnostic role of ctDNA in SCLC. Moreover, two studies have recently demonstrated that multicancer blood tests (with multi-gene or methylation analysis, respectively) could detect a broad range of cancer types, SCLC included [78,79]. CTC enumeration could be a more accurate, even if more expensive, strategy for early SCLC diagnosis. About a half of patients with LS-SCLC present detectable CTCs with an assay based on immunomagnetic enrichment and immunocytochemistry, with no CTC detectable with the same method in healthy donors [23]. By means of CellSearch assay, 3% of healthy cases [80] and 60–85% of patients with LS-SCLC may present detectable CTCs (Table S3). Preliminary data brace a potential application of LB also in the differential diagnosis between SCLC and NSCLC or other carcinomas. More abundant CNV changes and mutations, also with higher AFs, have been detected in ctDNA of patients with SCLC than with other tumor types, even with NSCLC [12,27]. This could reflect the higher proliferation rate with a tendency to early hematogenous spread of SCLC. In addition, the enumeration and phenotyping of CTCs [76,81] could suggest a diagnosis of SCLC rather than other cancer types.

### 4.2. Genomic Profiling

Different authors demonstrated that genomic characterization of SCLC is feasible by LB, with high detection rates for ctDNA (>90%) and CTCs (median of 85%). Analysis of ctDNA could provide both qualitative and quantitative data on tumor-related genomic alterations. A good concordance with tissue-based profiling has been reported with the sequencing of ctDNA by different groups [28,30,82]. As expected, *TP53* and *RB1* have been the most frequently altered genes detected on ctDNA [12,25,26]. Recurrent amplification of *SOX2, MYC* family genes, or *FGFR1* has also been reported by LB (Table 2), consistently with previous tissue-based analysis [83,84]. The differences in gene alteration rates reported by different authors (Table 2 and Table 3) may be related to limited sample sizes, to the variety of assays, gene panels, and depth of sequencing, to differences in tumor burden, ongoing therapies, or timing of sample collection (for example, alterations in *APC* and *AR* genes have been reported more frequently in relapse samples [52,54]), and to the possibility of hematopoietic variants (clonal hematopoiesis of indeterminate potential—CHIP) being misclassified as tumor derived [28,31,85]. Moreover, loss of heterozygosity events is difficult to detect at low AFs in ctDNA with common assays, and this could justify the lower frequencies of *TP53* or *RB1* deletions compared to tissue sequencing in previous studies [83]. In some cases, ctDNA analysis could reveal genomic alterations not detected in tissue, mirroring the spatial heterogeneity of the tumor [28]. One group performed single-cell sequencing of CTCs from SCLC patients, demonstrating that also CTCs constitute a reliable source for genomic profiling [75]. Moreover, this sort of information is not yet directly useful in the clinic today. SCLC has historically been treated as a homogenous cancer with no targetable driver genetic alterations. Recent data are diametrically changing this paradigm toward a critically heterogeneous disease, with the identification of different transcriptional subtypes [86] and also a variety of subpopulations of cells emerging with treatment resistance [87,88]. Such heterogeneity limits the reliability of tissue biopsies, enhancing the potential role of both ctDNA and CTCs, which could help a rational development of new therapeutic options [89,90].

### 4.3. Predictive Value

We found several studies reporting a correlation between tumor burden modifications in response to chemotherapy and quantitative changes in ctDNA, both in terms of ctDNA abundance and in terms of AF of specific gene mutations [10,12,28,33,34,82]. Moreover, specific qualitative molecular features have been associated with chemosensitivity (*APC* alterations) and chemoresistance (*TP53*, *ATM*, and *FLCN* alterations), respectively, but larger-scale studies are required to validate these findings [35]. A negative predictive value of detectable ctDNA at baseline has been reported for patients treated with atezolizumab. The reason is not clear, but higher levels of ctDNA may reflect a higher tumor burden, which has been already linked with the lower efficacy of immune-checkpoint inhibitors in lung cancer [91]. Even if these studies are based on small cohorts of patients, all together, these data support ctDNA monitoring in patients with SCLC as a potential tool for better management of the disease. Moreover, the best frequency of blood collection is not yet determined, and prospective studies are necessary to fully assess the reliability of ctDNA for clinical decisions. Moreover, predictive biomarkers for targeted therapies are still lacking in SCLC, albeit initial data are emerging from clinical trials exploring targeted agents, and ctDNA analysis could be helpful in such cases [25].

The association between CTC enumeration and treatment response is less clear. Different groups reported data showing a reduction in CTCs after treatments in SCLC patients [36,40,41,54]. Such reduction was also documented in other cancer types [92]. Moreover, no statistically significant correlation between the type of response and CTC count (both at baseline and after chemotherapy, assessed by CellSearch) was observed in studies by Naito, Hiltermann, Wang, Salgia, Aggarwal, and Belani [17,18,37,38,39,42]. Three different groups, on the other hand, observed significantly fewer CTCs among patients achieving a disease control compared to patients with PD as the best response. Moreover, these studies were conducted, respectively, in a small phase I cohort of patients, in a second-line setting with an antiangiogenetic agent, and in a mixed group of LS and ES-SCLC patients [36,45,58]. Different assays may detect different subpopulations of CTCs, which can show diverse behavior in the outflow from the tumor, not always mirroring the changes in the tumor size. For example, tumor cells down-modulate the epithelial markers undergoing epithelial mesenchymal transition (EMT); thus, CellSearch fails to capture these EpCAM-negative CTCs, while the OBP-401 assay for CTC identification, which is based on the telomerase expression, -could be more suitable for the detection of tumor cells involved in EMT process [46]. A Greek group widely studied CTCs with diverse phenotypes, and they observed a close relationship between changes in tumor burden and count of DLL3+, CD45− CTCs [49]. DLL3 has been known to be highly expressed in SCLC. More recently, Gay et al. described different transcriptional subtypes of SCLC, and DLL3 appeared to be more expressed in platinum-sensitive subtypes [86]. While the technology for CTC detection is not accessible in the majority of the laboratories, the novel identification of transcriptional subtypes and the development of chimeric antigen receptor (CAR) T cells or of antibody-drug conjugates may add new value to CTCs (i.e., for transcriptome analysis and for characterization of the proteins expressed on the surface of cancerous cells, the surfaceome).

### 4.4. Prognostic Value

The available data show an association between a higher quantity of cfDNA/ctDNA and shorter survival [12,28], with better outcomes in cases of reduction in ctDNA levels over therapy [51]. The prognostic significance of tumor mutational load, which appears higher in cases of mutations of cell cycle regulation genes [25], is not clear since conflicting data have been reported in small studies [25,28]. From a qualitative point of view, mutations of different genes have been associated with shorter OS by a single small study [27]. No other study reported such association, neither previous data derived from tissue analysis [93]. Mohan et al. reported a prognostic significance of mutations of any gene, included *TP53*, only when they are present in ctDNA with high AFs [10], suggesting a relationship with enhanced shedding of ctDNA rather than with specific genetic mutations. Anyway, the great heterogeneity in gene panels, depth of sequencing, and the quantity of blood collected and evaluated are major variables that prevent a reliable comparison among studies.

Several studies independently demonstrated a prognostic significance of CTC count in patients with SCLC. Moreover, the huge heterogeneity of these analyses did not allow us to reach a consensus on the optimal cut-off threshold of CTC number and on the time of plasma collection. One reason that could also hinder the achievement of a strict cut-off is the huge variability in the number of CTCs existing at baseline among ES-SCLC (median number: 9.5–91 per 7.5 mL), while the range appears less wide among LS-SCLC (median number: 1–21 per 7.5 mL) (Table S3). Baseline CTC count seems to be associated with the number of organs involved by disease [37,40,46,60,63], and in particular with liver metastases, but not with brain or bone metastasis [16,17,40,58]. Another hurdle could derive from the subpopulation of CTCs detected by a specific assay, as mentioned above. So the question may be if CTC numbers should be integrated by CTC phenotype as a criterion for prognostic definition and therapy decisions. Other prognostic information could derive from the enumeration of CTCs at different time points or from their longitudinal quantification, as demonstrated by different authors [16,17,18,36,37,59,60]. In our meta-analysis, a pooled HR for death of 2.63 was found considering the CTC population detected by CellSearch at baseline among SCLC patients at any stage. Anyway, a relevant risk of publication bias is suggested by the funnel plot (Figure S1). Moreover, the choice of ROC analysis for the identification of the optimal cut-off adopted in these studies creates a methodologic bias toward a higher HR. The highest HRs were reported by two studies assessing CTCs exclusively in patients with LS-SCLC [55,56], where the abundance of CTCs could reveal a micrometastatic disease. In 2014, Zhang and colleagues meta-analyzed results from seven studies and calculated a pooled HR of 1.9 for OS [94]. They found no publication bias; moreover, they included papers where CTCs were detected by different assays (five out of seven with CellSearch) and at different time points; furthermore, Normanno et al. [60] reported an HR with switched groups, but the value included in the meta-analysis was not adequately inverted [94].

### 4.5. Value of CTCs and ctDNA Changes in Small-Cell Transformation of NSCLC

**Figure 3:**
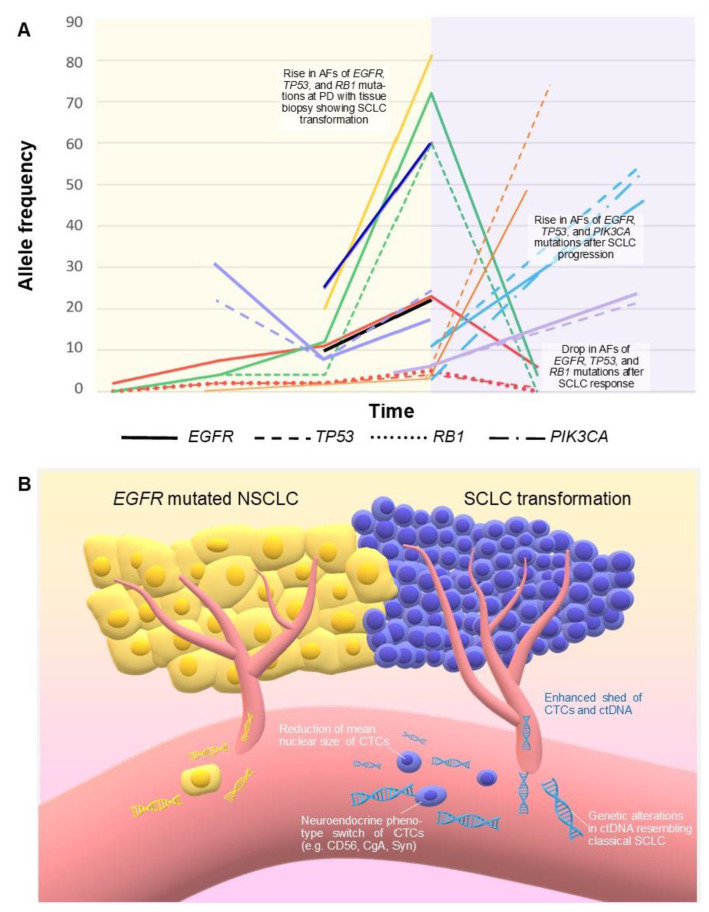
(**A**) Correlation between SCLC transformation of *EGFR* mutated NSCLC and changes in mutation allele frequencies over time detected by ctDNA in 9 patients, as reported in 7 evaluable studies (Table 3). Each patient corresponds to a different color. Time not in scale. Red: Schmid 2020 [69]; dark blue: Pizzutilo 2019 [64]; yellow and black: 2 patients by Minari 2018 [66]; green: Iijima 2018 [67]; orange: 1 patient by Tsui 2018, the other 2 patients were not reported because the diagnosis of histologic transformation was performed under chemotherapy potentially active against SCLC [70]; light blue: Mooradian 2017 [68]; purple: 2 patients by Vendrell 2020; a third patient was not represented because AF at the moment of transformation was not reported [71]. The yellow background represents the *EGFR* mutated NSCLC; the pink background represents the histologic transformation. (**B**) Enhanced DNA tumor shed and a higher number of CTCs have been reported in SCLC compared with NSCLC. These features, together with qualitative changes in ctDNA and CTCs, could also characterize transformed SCLC. *AF: allele frequency. CTCs: circulating tumor cells. ctDNA: circulating tumor DNA. PD: progressive disease.*

In some patients affected by *EGFR* mutated NSCLC, *EGFR* mutations become undetectable through LB after the failure of a TKI. In other cases, mutations in ctDNA could persist with similar or higher AF, even with secondary resistance mechanisms [95]. Among 67 patients with *EGFR* mutated NSCLC after PD to EGFR TKIs, and increased *EGFR* mutation abundance on ctDNA was evident in 37% of cases, while in the majority of cases, the allelic fraction of *EGFR* activating mutation was either stable (19%) or reduced (43%). Patients with stable or increasing *EGFR* mutant AF at PD showed worse PFS and OS [96]. Interestingly, increasing levels of *EGFR* activating mutation in ctDNA were described in all the case reports (8 patients overall) available in literature in which LB was performed after TKI failure with evidence of small cell transformation [64,65,66,67,69,71]. In two other cases, the *EGFR* activating mutation was not retained in neuroendocrine cells, or ctDNA analysis was performed after chemotherapies with platinum and docetaxel, and the *EGFR* mutant AF was not elevated [70]. Among the eight evaluable patients, the extent of the absolute increase in AFs of activating *EGFR* mutation at the moment of small cell transformation after TKIs, ranged from +2% up to +61%, with a median of +35% (Table 3, Figure 3). Transformed SCLCs usually retain original activating *EGFR* mutation, and the sharp increase in *EGFR* mutant AF at the moment of small cell transformation could be a consequence of the higher growth rate, the more aggressive behavior with increased tumor burden, and the enhanced DNA tumor shed observed in SCLC compared with NSCLC [12]. Typical CNAs observed in classical SCLC (Table 2) have also been reported in ctDNA in cases of histologic transformation, such as amplifications of *MYCL1*, SOX2, or *CNTN3* [70]. Furthermore, LB could allow a successful treatment with a third-generation EGFR TKI in those cases where the resistance mutation T790M appears on ctDNA after a previous small-cell transformation [72,73].

Marked higher counts of CTCs have been observed in SCLC in comparison with other malignancies, including NSCLC [15,81], and a correlation exists between CTC count and quantity of cfDNA [10,31]. In two studies, single-cell exome sequencing was performed in CTCs collected from patients presenting SCLC transformation. Mutations in *EGFR*, *PIK3CA*, *RB1,* and *TP53* were identified [75,76]. Zhu et al. demonstrated that a histological transformation can be reflected by a significant reduction in the mean nuclear size of CTCs, with phenotypic changes rapidly assessable with immunocytochemistry [76]. Given the continuous flow of CTCs, their “real-time” characterization may become a promising method for timely monitoring of the clonal evolution of solid tumors. Tissue re-biopsy appears now irreplaceable for detecting histologic transformation; furthermore, a ctDNA analysis could suggest a neuroendocrine transversion when it shows an increased *EGFR* muation AF, especially if together with typical SCLC-associated genetic alterations (Table 1 and Table 2), in patients with aggressive progression. CTC detection by morphology-based enrichment methods [81] could potentially become an alternative to tissue re-biopsy for detection of histologic transformation since these CTCs could undergo cytopathological analysis (i.e., positivity for CD56, CgA, or Syn in case of small cell carcinoma).

While these preliminary data deserve confirmations in wider studies, SCLC transformation may increase its frequency with more targeted anticancer treatments available, and its detection could be easier with the suggestions of an LB. The next step could be the design of studies dedicated to this subset of patients in order to expand their treatment options. Recently, the first clinical trial of durvalumab and olaparib for patients with *EGFR* mutated transformed SCLC has been launched (NCT04538378).

## 5. Conclusions

In conclusion, LB provides a huge amount of data in patients with SCLC, exploitable in diverse settings. Qualitative and quantitative changes in CTCs and ctDNA hold a diagnostic potential for both de novo and transformed SCLC, where a phenotypic switch of CTCs and changes in ctDNA could suggest a neuroendocrine transformation of NSCLC. In particular, ctDNA alterations (e.g., *EGFR* mutant AF) are easily evaluable also in non-academic laboratories. Despite this, a diagnosis of SCLC cannot rely only on LB findings. In addition, genomic profiling of SCLC is feasible by ctDNA analysis, even if results are affected by the adopted assay, tumor burden, or ongoing therapies. Such information may be precious in clinical trials exploring the targetability of molecular flaws. Monitoring response to treatment appears the most mature potential added value of ctDNA for the management of SCLC. CTCs hold a prognostic significance and a strong translational potential for SCLC, but the heterogeneity of available data and the less affordable technology for CTC detection in clinical laboratories hinder their implementation in present daily practice. Comprehensive integration of data derived from ctDNA and different CTCs subpopulations could provide accurate prognostic definitions, decisive biomarkers for therapy decisions, and a fertile field for translational research.

## Figures and Tables

**Table 1:** Most frequent (median throughout all studies ≥5%) mutated genes detected by ctDNA in 6 studies through different assays. Frequencies of genetic alterations researched in at least 2 studies are reported. N.: number of patients; Ref.: reference; -: not evaluated in gene panel.

Ref.	Herbreteau 2020 [29]	Mohan 2020 [10]	Owonikoko 2020 [25]	Devarakonda 2019 [26]	Du 2018 [27]	Almodovar 2018 [12]	Nong 2018 [28]
**Assay**	5 genes, QIAseq Targeted DNA custom panel	110 genes, custom panel	80 genes, custom panel with PlasmaSelect-R	54–73 genes, Guardant 360	127 genes, xGen Pan-Cancer Panel (AF >5%)	14 genes, custom panel with Resolution Bioscience targeted hybrid capture	430 genes, targeted deep sequencing, custom panel
**N.**	68	62	140	594	17	27	22
**Time of sample collection**	At relapse	At diagnosis	At relapse	Any	At diagnosis	Any	At diagnosis
**GENE**	**% mut**	**% mut**	**% mut**	**% mut**	**% mut**	**% mut**	**% mut**
TP53	65	79	86	72	24	67	91
KMT2D	-	13	-	-	76	-	-
RB1	51	32	58	18	24	37	64
SLIT2	-	8	-	-	-	-	27
MTOR	-	-	-	2	47	-	14
NOTCH1	6	13	15	6	53	15	9
ATRX	-	-	11	-	30	-	9
NF1	-	2	-	13	24	-	9
COLL22A1	-	13	15	-	-	-	-
CREBBP	-	5	13	-	-	-	18
BRCA2	-	2	-	6	24	-	18
TP73	-	10	14	-	-	-	-
EP300	-	8	8	-	29	-	14
APC	-	3	6	10	41	-	14
NOTCH3	8	5	9	-	-	11	14
ATM	-	-	-	3	35	-	9
ARID1A	-	0	-	12	53	-	5
AR	-	2	8	8	18	-	9
PIK3CA	-	5	4	8	-	11	14
PTEN	-	3	5	5	6	7	5
EGFR	-	2	2	14	18	-	5
PDGFRA	-	3	-	5	12	-	5
BRCA1	-	2	-	8	12	-	0

**Table 2:** Most frequent (median throughout all studies ≥5%) genes with copy number variation (CNV, namely amplification, and deletion) detected by ctDNA in 5 studies through different assays. Frequencies of genetic alterations researched in at least 2 studies are reported. The assay by Owonikoko could detect amplification events of *MYC* and *AURKA* only, but no results were reported by authors [25], then it is not included in the table. N.: number of patients; Ref.: reference; -: not evaluated in gene panel.

Ref.	Mohan 2020 [10]	Devarakonda 2019 [26]	Du 2018 [27]	Almodovar 2018 [12]	Nong 2018 [28]
**Assay**	Whole genome sequencing	54–73 genes, Guardant 360	Whole genome sequencing	14 genes, custom panel with Resolution Bioscience targeted hybrid capture	430 genes, targeted deep sequencing, custom panel
**N.**	62	594	24	27	22
**Time of sample collection**	At diagnosis	Any	At diagnosis	Any	At diagnosis
**GENE**	**% CNV**	**% CNV**	**% CNV**	**% CNV**	**% CNV**
RASSF1	55	-	58	-	-
SOX2	52	-	38	-	-
FHIT	58	-	29	-	-
FGF10	-	-	38	-	-
RB1	35	0	38	44	23
CNTN3	59	-	0	-	-
CCNE1	-	13	33	-	-
PIK3CA	-	23	0	30	-
CD274	20	-	25	-	-
MYCL	22	-	41	-	9
TP53	-	0	67	22	5
MYC	30	12	71	-	5
KIF2A	29	-	0	-	-
FGFR1	17	9	25	0	-
NFIB	23	-	0	-	-
MYCN	10	-	21	0	5
KIT	3	3	0	15	-

**Table 3:** ctDNA and CTCs changes in cases of SCLC transformation of *EGFR* mutated or *ALK*-rearranged NSCLC. ABS: abstract; NA: data not available. PD: progressive disease. tSCLC: transformed SCLC.

REF.	N.	Assay	Results
**ctDNA**			
Vendrell 2020 [71]	3	ddPCR and NGS	In 2 patients, elevation of AF in ctDNA of *EGFRdel19* (from 6% to 17%) and *TP53*_M246K_ (from 6% to 24%), and of *EGFRL858R* (from 4% to 6%) and *TP53*_L194R_ (from 3% to 6%), respectively, concurrent with evidence of tSCLC. Not available AF at the moment of transformation for the third patient. In all patients, the levels of the *EGFR* mutations in terms of copies/mL of plasma raised with SCLC progression.
Schmid 2020 [69]	1	NGS (Geneseeq Prime 425-gene)	Elevation of AF in ctDNA of *EGFRdel19* (from 0% to 23%), T790M (from 2% to 18%), *RB1*_Q850X_ (from 0% to 5%), and *TP53*_M237I_ (from 0% to 4%) concurrent with evidence of t SCLC. Subsequently, a reduction in AFs of these mutations was achieved with cisplatin-etoposide+RT.
Pizzutilo 2019 [64]	1	ddPCR (*EGFR*)	Elevation of AF in ctDNA of *EGFRdel19* (from 25% to 60%) with reduction in T790M/del19 Ratio (from 0.24 to 0.02) and detection of C797S concurrent with evidence of tSCLC.
Minari 2018 [66]	2	ddPCR (*EGFR*)	Elevation of AF in ctDNA of *EGFRdel19* (from 10% to 22%) and of *EGFRL858R* (from 20% to 81%), respectively, concurrent with evidence of tSCLC in 2 patients.
Iijima 2018 [67]	1	NGS (43 genes)	Elevation of AF in ctDNA of *EGFRdel19* (from 12% to 72%) and TP53_F134fs_ (similar AF) concurrent with evidence of tSCLC. Subsequent carboplatin-etoposide treatment led to a drop in AFs.
Tsui 2018 [70]	3	Targeted NGS and WGS	2/3 retained *EGFR* activating mutation after transformation in ctDNA and tissue, 0/3 presented T790M. Elevation in AFs of *EGFR* concurrent with evidence of PD of SCLC. *TP53* mutation was present before transformation and increased in 3/3 patients with PD of SCLC, together with the emergence of CNAs of genes such as *MYCL1*, *SOX2, SOX4*, and *EGFR.*
Nishioka 2018 [73]	1	NA	Evidence of *EGFR* T790M mutation in ctDNA after treatment for tSCLC, leading to successful therapy with osimertinib.
Mooradian 2017 [68]	1	NGS (Guardant360)	Elevation of AF in ctDNA of *EGFRdel19* (from 11% to 46%), TP53_V173L_ (from 11% to 55%), *PIK3CA*_E726K_ (from 3% to 51%), and *PIK3CA*_E545K_ (from 3% to 54%), concurrent with evidence of PD of tSCLC.
Ou 2017 [74]	1	NGS (FoundationACT)	After PD to 2° line lorlatinib in a patient with *ALK* rearrangement, ctDNA analysis showed persistence of *ALK* rearrangement (estimated AF 30–45% vs. 40–54% before lorlatinib) and disappearance of acquired G1202R, concurrent with SCLC transformation.
Alì 2016 [77]	1	PCR	Evidence of *EGFR* T790M in ctDNA concurrent with transformed SCLC in tissue biopsy harboring *EGFR* activating mutation, but not T790M.
Piotroska 2015 [65]	1	Beaming (EGFR)	Increasing levels of *EGFR* activating mutation, with T790M levels remaining suppressed, at the time of progression with SCLC transformation.
Han 2017 [72] **ABS**	11	NGS	3/11 patients developed *EGFR* T790M mutation in the post-transformation ctDNA rather than in their tissue samples.
**CTC**			
Zhu 2020 [76]	14	Aptamer-modified PEG-PLGA-nanofiber microfluidic system for CTC capture, and single-cell sequencing	Histological transformation was reflected by CTC phenotype change from TTF1+, NapsinA+, CK7+, P63- toward CD56+, CgA+, and Syn+, with a significant reduction (*p* < 0.05) of the mean nuclear size of CTCs. 14/14 patients showed the same molecular characteristics for *EGFR*, *RB1*, and *TP53* between CTC and tissue samples.
Ni 2013 [75]	1	CellSearch and Single-Cell Exome Sequencing in CTC	*EGFR* del19 was identified in tSCLC biopsy as well as in CTCs. *PIK3CA*, *RB1*, and *TP53* mutations were identified in tSCLC tissue biopsy and CTCs, with higher abundance than in the original NSCLC tissue.

## Supplementary Materials

The following are available online at https://www.mdpi.com/article/10.3390/cancers13092265/s1. Figure S1: Prognostic significance of CTCs assessed by CellSearch in patients with SCLC at baseline: funnel plot showing hazard ratios and standard errors for overall survival; Table S1: Studies of liquid biopsy in de novo SCLC, included in the systematic review, with diagnostic, genomic profiling, predictive and/or prognostic findings. Studies are classified in three groups on the basis of the tumor-derived component analyzed (CTCs, ctDNA, or both); Table S2: Detection of ctDNA in SCLC. Table S3: Detection of CTCs in SCLC at baseline; Table S4: Studies included in meta-analysis of prognostic value of baseline CTCs assessed by CellSearch.
