# Supplementary material for: Liquid Biopsy for Small Cell Lung Cancer either De Novo or Transformed: Systematic Review of Different Applications and Meta-Analysis
Source: Cancers (Basel). 2021 May 8;13(9):2265. doi: 10.3390/cancers13092265 (PMC8125928; doi:10.3390/cancers13092265)
Supplement: Supplementary file 1 [file cancers-13-02265-s001.zip › supplementary.pdf]

cancers

MDPI

Supplementary materiel

Liquid Biopsy for Small Cell Lung Cancer either *De Novo* or Transformed: Systematic Review of Different Applications and Meta-Analysis

Elio Gregory Pizzutilo, Martino Pedrani, Alessio Amatu, Lorenzo Ruggieri, Calogero Lauricella, Silvio Marco Veronese, Diego Signorelli, Giulio Cerea, Laura Giannetta, Salvatore Siena and Andrea Sartore-Bianchi

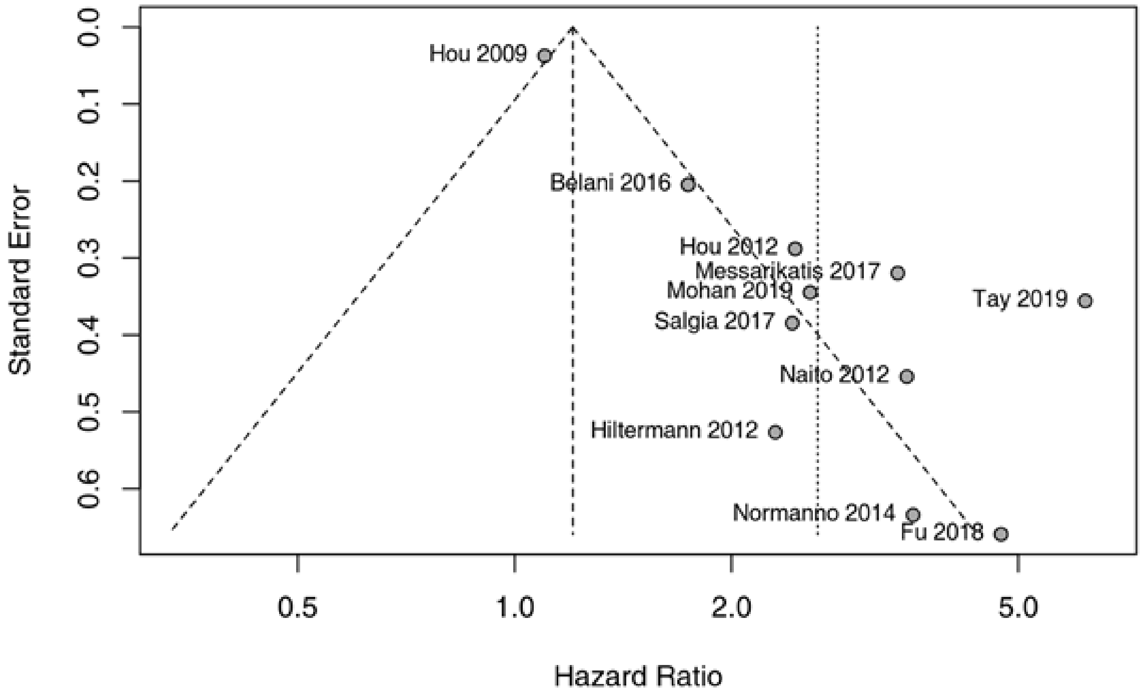

Figure S1. Prognostic significance of CTCs assessed by CellSearch in patients with SCLC at baseline: funnel plot showing hazard ratios and standard errors for overall survival.

Table S1. Studies of liquid biopsy in *de novo* SCLC, included in the systematic review, with diagnostic, genomic profiling, predictive and/or prognostic findings. Studies are classified in three groups on the basis of the tumor-derived component analyzed (CTCs, ctDNA, or both).

REF. Country	Type	N. (ED/ LD)	Treatment	Assay	Main Findings
			**CTCs**		
Wang 2020 [63] China	Prosp	138 (NR)	PE, 1st line	CEP8+, CD45- FISH	**Prognostic Findings:** nomograms incorporating baseline-CTCs, age, staging, NSE and treatment predicted different OS among different risk groups (p<0.0001)
Tay 2019 [55] UK	Prosp	75 (0/75)	CRT	CS	**Prognostic findings:** Baseline CTC was an independent prognostic factor for both OS and PFS in MVA. mOS in <15 vs ≥15 CTCs was 26.7 m vs 5.9 m (p=0.001). In patients

					with >14 CTCs (17/75, 23%), survival was limited to 1 year in 70% and 2 years in 100% of patients.
Messaritakis 2019 [49] Greece	Prosp	108 (71/37)	1st line PE or CRT	CS IF (DLL3+, CD45-)	**Predictive Findings:** One-treatment cycle significantly decreased both the detection rate (p<0.001) and the absolute number (p<0.001) of DLL3+/ CD45− CTCs, while on disease progression, they were both increased compared to post-1st cycle values (p<0.001). **Prognostic Findings**: detection of DLL3+/CD45- CTCs at baseline was associated with decreased PFS (MVA HR=10.8; p=0.005) whereas their detection after 1st cycle was associated with decreased OS (MVA HR=28.2; p=0.016).
Su 2019 [32] China	NR	48 (40/8)	1st line PE	CS and single-cell sequencing	**Genomic Profiling:** copy number losses in *TP53* and *RB1* were identified in 65% and 81% of the patients, and losses in chromosome 4 and chromosome 5q in 56% and 38%, respectively. Among 10 patients with paired samples, 68%–99% of mutations observed in tissues were detectable by CTC sequencing. CTCs shared the majority of the mutations with the primary tumor, while metastatic sites formed minor clones unobserved in CTCs. **Predictive Findings:** Using a CNA score (based on 10 CNA regions) from single-cell sequencing of CTCs collected before treatment, 20 of 25 chemorefractory patients were correctly identified as having a high score, and 15 of 16 chemosensitive patients were corrently identified as having CNA scores below 0 (p<0.0001,2-tailed Fisher exact test). **Prognostic Findings:** Such CNA score could predict PFS and OS. Low CNA-CTC scores (<0) had significantly prolonged PFS and OS (PFS p=0.0042; OS p=0.0006). High CNA score on MVA independently predicted poor PFS (p<0.001) and OS (p=0.00072).
Gadgeel 2018 [62] USA	Phase II	45 (45/0)	Pembrolizumab maintenance after 1st line PE	CS	**Prognostic Findings**: PFS and OS did not correlate with the pre-maintenance CTC count or with changes in the CTC count during therapy with pembrolizumab.
Fu 2018 [56] China	Retro	112 (0/112)	CRT	CS	**Prognostic Findings:** The number of CTCs and the WBMTV at baseline were independent relevant factors for PFS and OS in MVA, but not the CTC count after 1 or 4 cycles of chemotherapy. More than 19.5 CTCs per sample at baseline correlated with worse PFS (15 vs 21 mo, p=0.001) and OS (23 vs 28 mo, p<0.001) in patients with similar tumor volume by use of FDG-PET. Patients were classified according to CTC count and WBMTV in 4 subgroups able to discriminate both PFS and OS.
Pietanza 2018 [43] USA	Phase II	94 (94/0)	2nd or 3rd line temozolomide + veliparib or placebo	CellSearch	**Predictive Findings**: a zeroing in CTC count was observed in those patients achieving a PR and a rise of CTC enumeration at progression. **Prognostic Findings**: Pre-treatment CTCs >5, were associated with worse OS (5.6 versus 9.7 months p=0.001) in UVA. A persistently elevated CTC number >5 at cycle 2 was associated with worse OS in UVA (7.2 vs 8.8 mo, p=0.012).

Messaritakis 2018 [44] * Greece	Phase II	56 (NR)	2nd line pazopanib	CS, IF (CKs, Ki67, M30, Vim)	**Predictive Findings**: Patients with disease control (PR/SD) had a significantly lower number of CTCs compared to patients with PD at all evaluated time points (pre-treatment, after one pazopanib cycle and on PD) as assessed by CS (PD median of 17 CTCs/7.5 ml; PR median of 0 CTCs/7.5 ml and SD median of 2 CTCs/7.5 ml, respectively; p=0.006). Clinical response to pazopanib was not associated with the presence of any subpopulation of CTCs.
Koinis 2017 [45] * Greece		58 (43,15)		CS	One pazopanib cycle significantly decreased the number of CTCs as detected by CS (p=0.043), as well as by IF CK+/Ki67+ (p<0.001), CK+/M30+ (p=0.015), and CK+/ Vim+ (p<0.001). On disease progression CTC number was significantly increased (CS, p=0.027; CK+/Ki67+, p<0.001; CK+/M30+, p=0.001 and CK+/Vim+, p<0.001).<br>**Prognostic Findings**: Patients with >5 CTCs at baseline had significantly shorter PFS (p<0.001) and OS (p<0.001). The median OS was significantly higher in patients without detectable CTCs after one pazopanib cycle compared to patients with detectable CTCs (p=0.036). Detection of CK+/Vim+ CTCs after one treatment cycle (HR: 7.9, p<0.001) and CTC number on disease progression (HR 2.0; p=0.005) were associated with decreased OS in MVA.
Messaritakis 2018 [57] ** Greece	Prosp	66 (40/26)	1st line PE or CRT	CS, IF(Bcl2+,CD45-)	**Prognostic Findings**: CTCs by CS and by Bcl-2+/CD45− IF at baseline were associated with a decreased PFS (HR: 5.9, p=0.01; HR: 4.5, p=0.005) in MVA.<br>≥5 CTCs by CS at baseline and on PD and detectable CTCs by Bcl-2+/CD45− IF at baseline and after one treatment cycle were associated with decreased OS (HR: 4.9, p=0.031; HR: 3.8, p=0.009; HR: 4.3, p=0.001; HR: 13.9, p=0.007, respectively), in MVA.
Messaritakis 2017 [36] ** Greece		108 (71/37)		CS, IF (CD45-, TTF1+ or CD56+)	**Predictive Findings**: the number of CTCs by CS both at baseline and after one treatment cycle was higher in the group of patients who experienced a PD compared to patients with no PD (54.2% vs 6.0%; p=0.004 and 29% vs 0.0%; p=0.022); however, using IF this difference could not reach any statistical significance.<br>**Prognostic Findings:** ≥5 CTC by CS at baseline was an independent factor for PFS (p=0.048) in MVA, and for reduced OS (p<0,001) in UVA. Only ≥5 CTCs at the time of PD was prognostic for OS (p=0.041) in MVA. Different CTC subpopulations by IF were not associated with PFS (TTF1+/CD45−, CD56+/CD45− and TTF1+/CD56+) either at baseline or after one treatment cycle.
Aggarwal 2017 [37] USA	Prosp	50 (30/20)	1st line PE or CRT	CS	**Predictive Findings**: CTCs at baseline and a decrease in CTCs with chemotherapy were not related to response (Kruskal-Wallis Test p=0.61).<br>**Prognostic Findings:** baseline 50 CTC threshold significantly correlated with both OS (p=0.0116) and PFS (p=0.0002), while the baseline 5 CTC threshold did not. At

					cycle 2, 5 CTC threshold correlated with PFS (p<0.001) and OS (p=0.0001), even in the ED subgroup.
Carter 2017 [50] UK	Prosp	18 (18/0)	1st line PE	CS and whole-genome sequencing	**Predictive Findings**: CNA signature measured in CTCs from pre-treatment blood samples correctly classified 83.3% of the cases as chemorefractory or chemosensitive. **Prognostic Findings**: A significant difference was observed in PFS (HR 0.42, p=0.04), not in OS (HR 0.55, p=0.15), between patients designated as chemorefractory or chemosensitive.
Shen 2017 [47] China	Prosp	80 (77/3)	1st line PE	LT-PCR	**Predictive Findings**: Patients with PR or SD presented higher baseline CTC counts than patients with PD (p=0.0365). A reduction of CTC enumeration after two cycles of chemotherapy was significantly correlated with PR (p=0.0380), instead of SD (p=0.4934). **Prognostic Findings:** Patients with positive or negative CTC count at baseline had similar PFS (p= 0.625). Patients with relative low CTC level had significantly longer PFS than those with high CTC level (p=0.0458) and a trend for longer OS (p=0.056).
Salgia 2017 [42] USA	Phase II (post-hoc)	61 (61/0)	1st line PE +/- CXCR4 peptide antagonist	CS and anti-CXCR4	**Predictive Findings**: None of the biomarkers was predictive of treatment response **Prognostic Findings**: CTCs ≥6 were prognostic of shorter PFS and OS at baseline (p=0.024; p=0.017) and at cycle 2, day 1 (p=0.001; p=0.001). Baseline CXCR4+ CTCs ≥7% was prognostic of shorter PFS (p=0.029) but not for OS.
Wang 2017 [23] China	Prosp / case-control	42 (25/17)	1st line PE	Immuno magnetic and IHC (CD45-, panCKs+)	**Diagnostic Findings:** CTCs were found in 86% of 42 patients, including 17 with LS-SCLC. No CTCs was detected among 20 healthy donors or cases with benign lung disease**.** **Prognostic Findings**: Both baseline CTC number (≥2CTC) and the change in CTC number after 1 cycle of chemotherapy were significant prognostic factors for PFS in UVA (p=0.0012, p<0.001, but not in MVA (p>0.05).
Pietanza 2016 [58] USA	Phase I	14 (14/0)	1st line PE + sonidegib	CS	**Predictive Findings:** 10 of 11 patients with a PR had CTC = 0 at time of first follow-up scan, as compared to none of the 3 patients with SD. At PD, 5 of 13 patients had a rise in the CTCs. **Prognostic Findings:** baseline CTC >200 was prognostic for OS (6.2 vs 25.7 months). Persistently elevated CTC number at cycle 2 day 1 was associated with worse OS in UVA (25.0 months if CTC=0 vs 5.5 months for CTC≥1).
Belani 2016 [38] USA	Phase II	120 (120/0)	1s line PE +/- vismodegib or cixutumumab	CS	**Predictive Findings:** test for an association with CTC baseline count and response was not significant (p=0.17). **Prognostic Findings**: The adjusted OS HR comparing high (>100) and low CTC count was 1.74 (1.17-2.61; p=0.006) with median OS of 7.2 vs 10.5 months; PFS HR comparing high and low groups was 1.69 (p=0.01; 1.13-2.52).
Wang 2016 [39] China	Prosp	96 (32/64)	1st line PE or CRT	CS	**Predictive Findings:** No association between any threshold of CTC count at baseline and type of treatment response.
Cheng 2016 [59]	Prosp	89 (89/0)	1st line platinum based	CS	**Prognostic Findings:** a cut-off of <10 CTCs was prognostic at baseline for longer OS (16.6 vs 8.2 months, MVA HR

China					0.304, p<0.0001), after the second cycle of chemotherapy for OS (12.7 vs 6.9 months, MVA HR 0.295, p=0.0002) and PFS (3.1 vs 5.6 months, MVA HR 0.467, p=0.0211), and at disease progression for OS in UVA (8.3 vs 13.1 months, p=0.0053). Combining the CTC count at baseline and after second cycle and RECIST response, an algorithm was created which resulted more accurate to evaluate prognosis.
Normanno 2014 [60] Italy	Prosp	60 (60/0)	1st line PE	CS	**Prognostic Findings**: OS HR for multiple CTC baseline cut offs: <50 CTCs: HR 0.63 (0.34-1.18), <2CTCs: HR 0.28 (0.08-0.96), <8CTCs: HR 0.37 (0.16-0.87), <282 CTCs: HR 0.5 (0.26-0.95). A strong reduction of CTC count after one cycle of chemotherapy (>89%) was deeply associated to lower risk of death (HR 0.24, 95% CI 0.09–0.61, p=0.02).
Igawa 2014 [46] Japan	Prosp	30 (22/8)	1st line PE	OBP-401	**Predictive Findings:** Among the patients who exhibited a PR following two cycles of chemotherapy, the mean CTC count tended to increase (2.32 cells/7.5 ml) compared with the mean CTC count at baseline (0.84 cells/7.5 ml) regardless of a reduction in tumor volume (p=0.05). **Prognostic Findings**: Among the patients that achieved a PR, who had <2 CTCs after two cycles of chemotherapy tended to have a longer PFS (8.3 vs 3.8 months, p=0.07). Baseline >2 CTCs was prognostic in MVA for OS: 14.8 vs 3.9 months (p=0.007), OS HR 3.91 (p=0.026).
Huang 2014 [61] USA	Prosp	25 (25/0)	1st line PE	CS	**Prognostic Findings:** Baseline CTCs and change in CTCs from baseline to post-treatment were not statistically significantly associated with survival, but trended toward significance.
Shi 2013 [48] China	Prosp	55 (28/27)	1st line PE or CRT	CK-19 mRNA-positive CTC by Rtq-PCR	**Predictive Findings:** tumor response was significantly associated with the reduced detection rates of CTCs after 1 (p=0.008) and 3 cycles (p<0.001), with a trend towards higher CTCs at baseline among patients achieving SD/PD vs PR/CR(p=0.059). **Prognostic Findings:** detectable CK-19 mRNA-positive CTCs at baseline, after 1 and 3 cycles, were independent predictors of PFS (HRs: 2.92, 3.15, 3.22) and OS (HRs: 2.65, 3.28, 3.31) in MVA
Hiltermann 2012 [18] Netherlands	Prosp	59 (38/21)	1st line PE or CRT	CS	**Predictive Findings:** The decrease in CTCs after one and four cycles of chemotherapy and the absolute CTC number after one cycle of chemotherapy did not correlate with tumor response. **Prognostic Findings:** <2 CTCs correlated with improved PFS and OS (p≤0.001) in MVA after one cycle of chemotherapy (OS HR 7.9 p≤0.001), but not at baseline. Lack of measurable CTCs at baseline (27% of patients) was associated with prolonged OS (HR 3.4; p≤0.001). Patients with CTC<2 (n=27) after four cycles of chemotherapy had longer PFS (p=0.007) and OS (p=0.05).
Naito 2012 [17] Japan	Prosp	51 (24/27)	1st line platinum based or CRT	CS	**Predictive Findings:** No significant difference between the CR/PR subsets and SD/PD subsets in the baseline CTC count (median 4 [range, 0–1683] versus 4 [range, 0–5648]) or post-treatment CTC count (0 [0–44] versus 0.5 [0–253]).

					No significant differences among CR, PR, SD and PD groups in post-treatment CTC reduction. **Prognostic Findings:** Threshold of 8 CTCs was prognostic for OS at any time point: baseline (8.5 vs 17.2 months, HR, 3.50; 1.45–8.60), post-treatment (4.1 vs 13.9 months, HR 2.76 0.97– 6.92, p=0.0562) and relapse (4 vs 11.8 months, HR 6.20 2.39 –17.52, p=0.0002) in MVA.
Hou 2012 [16] UK	Prosp	97 (66/31)	1st line platinum based or CRT	CS	**Prognostic Findings:** ≥50 CTCs at baseline and after one cycle of chemotherapy were independent prognostic factors on MVA for both PFS (HR 2.01, 1.17-3.46, p=0.011 and 2.45, 1.39-4.30, p=0.002) and OS (HR 4.20, 1.44-12.25, p=0.008 and HR 5.49, 1.78-16.91, p=0.003).
Hou 2009 [40] UK	Prosp	88 (53/35)	1st line platinum based or CRT	CS	**Prognostic Findings:** The median OS for patients with >300 CTCs (highest quartile) was 134 days vs 443 days for patients with <2 CTCs (lowest quartile) (p=0.005). CTC number at baseline was prognostic for OS by UVA (HR 1.1, 1.02–1.18, p=0.015), but not on MVA. Persistently elevated CTC number (HR 1.43, 1.09–1.86, p=0.01) at day 22 was an adverse prognostic factor in UVA.
Bevilacqua 2009 [41] Italy	Case series	5 (5/0)	1st line PE or BSC	CS	**Predictive Findings:** 2 patients who achieved a PR presented also a significant reduction of CTCs following therapy.
Kularatne 2002 [54] UK	Prosp	11 (3/8)	Ifosfamide +PE	Flow cytometry (Ber EP4+) and magnetic bead isolation	**Prognostic Findings:** A general reduction of CTCs at the end of treatment compared to baseline is reported. One patient who was still alive at 44 months with initially elevated CTC levels, responded well to treatment, with a steady reduction in CTCs by the end of the study.
**CTC and ctDNA**					
Mohan 2020 [10] UK	Retro / case-control	69 (30/39)	1st line PE or CRT	CS<br>Genome-wide and targeted cfDNA sequencing	**Diagnostic Findings**: tumor related CNA were detected in 84% (58/69) of all SCLC samples; 93% of ES-SCLC and 77% of LS-SCLC (with statistically significant differences in CNA metrics between ES and LS), but in none of the 16 non-cancer controls. Different fragment size of cfDNA among LS, ES, and non-cancer controls. At least one non-synonymous somatic mutation was detected in 94% of 62 patients (97% of 29 ES, 91% of 33 LS), but in none of the 23 non-cancer controls.<br>**Genomic Profiling**: potential therapeutic targets in >50% of patients using both a genome-wide and targeted cfDNA sequencing (panel of 110 genes) with the aim of a longitudinal monitoring of tumoral mutations and CNA.<br>**Predictive Findings:** In a group of 6 patients monitored with serial whole genome and targeted cfDNA sequencing, cfDNA changes predict partial responses in 4/6 cases and relapses in 4/6 cases.<br>**Prognostic findings:** cfDNA CNA readouts, TP53 VAF and highest VAF showed a significant positive correlation with baseline-CTC number (p=0.0001).<br>More than 7 CTCs correlated with worse prognosis in UVA, OS HR 2.57 [1.31, 5.06] p<0.001

					TP53 AF and highest AF of any gene predict shorter survival in UVA. CNA readouts and less than 5 mutations correlated with worse prognosis in MVA.
Feng 2020 [97] **ABS** China	Prosp	35 (35/0)	1st line PE	- not reported - NGS	**Genomic Profiling:** 78% of tissue derived mutations were observed in ctDNA. **Predictive Findings**: good concordance between molecular tumour burden index on ctDNA and imaging evaluation; no concordance with CTC
Aggarwal 2017 [31] **ABS** USA	Prosp	12 (12/0)	None	CS NGS of cfDNA	**Genomic Profiling:** in a cohort of 12 patients with relapsed/refractory SCLC, identical genetic alterations were identifiable from both CTC DNA and cfDNA. Conversely, limited concordance was found with tissue-based profiling, especially for genes with low AF. **Prognostic Findings:** no association was reported between CTC count at the time of PD and survival or disease stage at diagnosis.
**ctDNA**					
Herbreteau 2020 [29] France	Phase II	68 (68/0)	2nd line atezolizumab or chemotherapy	ctDNA NGS (custom panel 5 genes)	**Genomic Profiling**: gene mutations found in 71% of patients (in 25% at least 1 *NOTCH* mutation). **Predictive Findings**: patients treated with atezolizumab with detectable ctDNA had lower disease control rate (13% vs 50%, p=0.0145); no differences with chemotherapy. **Prognostic Findings**: mOS 7.6 months in the presence of a detectable mutation vs 13.3 months, independently from treatment (OS HR 2.60 (1.11–6.08) p=0.0272)
Owonikoko 2020 [25] USA-EU	Phase II	155 (155/0)	2nd line paclitaxel +/- alisertinib	ctDNA NGS (custom panel 80 genes)	**Genomic Profiling**: Genetic alterations of ctDNA were detected in 90% of 155 evaluable patients and the genomic profile was consistent with that previously identified in SCLC tumor tissue. **Predictive/Prognostic Findings**: a correlation between mutational load and OS, but not PFS, was evident in the alisertib arm. A marginal correlation with PFS or OS in the alisertib arm was found with some individual mutated genes (*RB1*, *ADCY1*, *CNTNAP2*, *ZNF217*, *BCLAF1*). A stronger correlation with better survival was evident with the alteration of genes involved in cell cycle regulation among patients treated with paclitaxel+alisertib compared with paclitaxel+placebo: PFS 3.68 versus 1.80 months [HR=0.395, 95% CI: 0.239–0.654, p=0.0003], OS 7.20 versus 4.47 months [HR=0.427, 95% CI: 0.259–0.704, p=0.00085]. Patients without mutations in cell cycle regulators had no improvement in survival.
Thomas 2019 [34] USA	Phase II	1	2nd-4th line Olaparib and durvalumab	ddPCR	**Predictive findings**: drop in mutation copies of TP53 R119L, BRCA1 E1580Q, and NFKB2 G826R in ctDNA in a patient with a complete response
Devarakonda 2019 [26] USA	Prosp	564 (NR)	None	ctDNA NGS (Guardant 360, 54-73 genes)	**Genomic Profiling**: The detection rate of mutations or amplifications was 91%, with higher AFs in samples collected at diagnosis, compared to relapse, while the number of alterations detected did not differ significantly. The authors highlighted the identification of potentially targetable alterations involving the androgen receptor

					gene (AR), the RTK/RAS pathway, or genes involved in DNA repair.
Zhang 2019 [35] China	NR	28 (13/15)	1st line PE or CRT	ctDNA NGS	**Predictive Findings**: distinct molecular features by ctDNA analysis in patients with chemosensitive or chemorefractory SCLC. Alterations of *TP53* (66.7% vs 6.2%), *ATM* (66.7% vs 12.5%), and *FLCN* (41.7% vs 0) were higher in the chemorefractory group and related with significantly shorter PFS, while *APC* abnormalities occurred more frequently among chemosensitive patients (16.7% vs 68.8%). Also CNAs of *SH2D5*, *CA12*, *LMNA*, *PTCH1*, and *LIG4* showed a differential pattern.
Almodovar 2018 [12] USA	Prosp	27 (16/11)	Different lines	ctDNA NGS (14 genes)	**Genomic Profiling**: 85% of serial plasma samples, collected at different time points from 27 patients, presented disease-associated mutations in cfDNA with AFs ranging from 0.1% to 87%. **Predictive Findings:** Studying serial plasma samples at different time points of their clinical history, they observed changes in cfDNA mutation abundance: a rise of AFs or copy number variations before evidence of disease progression and, conversely, a reduction in cases of disease response. **Prognostic Findings** a significant association was found between increased cfDNA genomic equivalents (GEs) and worse OS: MVA HR 2.73, 95% CI: 1.27–5.86, p=0.0099. 1-year survival was 90% for patients with 2000 cfDNA GEs, with progressive declining up to 13% in patients with 16000 GEs.
Du 2018 [27] USA	NR	24 (13/11)	1st line PE and/or CRT	ctDNA WGS + targeted NGS	**Genomic Profiling**: copy number abnormalities in 67% (16/24) and mutations in all cases evaluated (17/17). **Prognostic Findings**: significant association with survival of mutations in *SETBP1* (OS HR 4.785, PFS HR 3.009), *PBRM1* (OS HR 3.532, PFS HR 3.029), *ATRX* (OS HR 4.024), *EP300* (OS HR 3.382), *PIK3CA* (OS HR 0.3034) and in *ATM* gene (PFS HR 4.604). Summing up the number of mutations, a mutation risk index was strongly associated with poor OS/PFS.
Nong 2018 [28] China	Prosp	22 (11/11)	1st line platinum based	ctDNA NGS (custom panel 430 genes)	**Genomic Profiling**: they reported a median of 16 mutations per sample with a high concordance with results from tissue analysis (median of 94% of paired mutations, by deep sequencing), showing also a correlation in allelic frequency between plasma and tissue. Some mutations was exclusively detected in ctDNA, reflecting spatial heterogeneity of SCLC. Furthermore, comparing samples collected at baseline and after treatment, new mutations appeared or became dominant in in the post-treatment samples. **Predictive Findings**: changes in ctDNA levels reflected changes in tumor burden on imaging, with average VAF of main mutations decreasing after platinum therapy and with other clones rising at disease progression. **Prognostic Findings**: there was no correlation with total mutation burden, mutations of specific genes, or AF of

					certain mutations and survival. Conversely, high ctDNA level significantly correlated with shorter PFS (5.3 vs 10 months; MVA HR 8.4, p=0.008) and OS (9.3 vs 25.0 months; MVA HR 4.7, p=0.021).
Fernandez-Cuesta 2016 [11] Russia	Case-control	51 (9 st. IV, 28 st. III, 14 st. I-II)	None	cfDNA PCR	**Diagnostic Findings**: *TP53* mutations identified in cfDNA of 49% SCLC patients (35.7% of those with early-stage), and in 11.4% of 123 non-cancer controls.
Board 2008 [24] UK	Case-control	10 (5/5)	None	cfDNA RT-PCR	**Diagnostic Findings**: higher DNA concentration in plasma (not in serum) of SCLC patients compared to 10 healthy volunteers (24.5 ng/mL versus 5.1 ng/mL, p=0.002), with higher proportion of longer fragments (272 bp vs 60 bp).
Gonzales 2000 [33] Spain	Prosp	35 (11/24)	1st line PE or CRT	PCR (microsatellite markers and *TP53* mutations in cfDNA)	**Predictive Findings:** correlation between clearance, reappearance, or persistence of plasma DNA alterations (microsatellite modifications and *TP53* mutations) with response, disease recurrence, and no response to treatment, respectively. **Prognostic Findings**: longer OS in patients with concomitant microsatellite modifications and *TP53* mutations.
Ying Jin 2020 [52] **ABS**	NR	58 (23/35)	1st line PE or CRT	NGS (1021 genes)	**Genomic Profiling:** recurrent genes were *TP53* (86%), *RB1* (57%), *LRP1B* (34%), *CREBBP* (26%), and *MLL3* (22%). Median blood-based tumor mutation burden (bTMB): 7.9 [0-26], and median clonal bTMB: 7 [0-25], higher in ED pts (p=0.019 and p=0.041, respectively). **Prognostic Findings**: prolonged PFS in pts with higher clonal bTMB (p=0.016, HR 0.37) in LD pts. *PIK3CA* mutations was associated to shorter PFS (p=0.001, HR 0.11). In ED pts, *NOTCH1* gene wild type was linked with longer PFS (p=0.036, HR 0.38).
Yaung 2019 [30] **ABS** USA-Germany	Retro	24 (NR)	1st line PE or CRT	ctDNA NGS	**Genomic Profiling**: a high concordance between somatic mutations was found in pre-treatment ctDNA and in matched SCLC tissue. Conversely, about a half of the mutations detected in post-treatment samples, with a median of 4 single nucleotide variants (SNVs), were not present at baseline.
Yaung 2018 [53] **ABS** USA-Germany	Prosp	56 (56/0)	1st line PE or CRT	ctDNA NGS	**Prognostic Findings**: correlation between higher genomic heterogeneity (in terms of quantity and abundance of somatic variants) of SCLC measured by ctDNA sequencing and longer OS.
Palma 2018 [51] **ABS** USA-Germany	Prosp	72 (NR)	1st line	AVENIO ctDNA 197-gene NGS	**Prognostic Findings**: a survival benefit was observed among patients with a continuous drop in ctDNA levels (as mutant molecules per milliliter-of-plasma) during chemotherapy (OS HR 1.9).

* and ** = studies evaluating partially the same population of SCLC patients. ABS: abstract; CNAs: copy number aberrations; CRT: chemoradiotherapy; CS: CellSearch; CTCs: circulating tumor cells; ctDNA: circulating tumor DNA; IF: immunofluorescence; LT-PCR: ligand-targeted PCR; MVA: multivariate analysis; NGS: next generation sequencing; NR: not reported; OS: overall survival; PD: progressive disease; PE: platinum, etoposide; PFS: progression free survival; PR: partial response;

Prosp: prospective; Retro: retrospective; SD: stable disease; UVA: univariate analysis; WBMTV: whole-body metabolic tumor volume.

Table S3. Detection of CTCs in SCLC at baseline. Pts= patients; DR: detection rate.

Ref.	N. Valuable pts	Assay	DR (at least 1 CTC/7,5 ml)	LD N pts	Median CTCs N. in LD	DR in LD	ED N pts	Median CTCs N. in ED	DR in ED
Hou 2009 [40]	50	CellSearch	86%	20	NA, median overall 28	NA	30	NA, median overall 28	NA
Hou 2012 [16]	97	CellSearch	85%	31	1	61%	66	NA, median overall 24	NA
Naito 2012 [17]	51	CellSearch	NA	27	1	N.A.	24	9.5	NA
Hiltermann 2012 [18]^	59	CellSearch	85%	21	21	67%	38	38	95%
Shi 2013 [48]*	55	CK-19 mRNA-positive CTC by Rtq-PCR	78%*	27	NA	59%	28	NA	96%
Normanno 2014 [60]	60	CellSearch	90%	0	-	-	60	47	90%
Huang 2014 [61]	25	CellSearch	96%	0	-	-	25	75	96%
Cheng 2016 [59]	89	CellSearch	88%	0	-	-	89	30	88%
Wang 2016 [39]	96	CellSearch	49%	64	NA		32	NA	
Aggarwal 2017 [37]	50	CellSearch	94%	20	1.5	NA	30	91	NA
Salgia 2017 [42]	78	CellSearch	83%	0	-	-	78	51	83%
Fu 2018 [56]	112	CellSearch	85%	112	19.5	85%	0	-	-
Tay 2019 [55]	75	CellSearch	60%	75	1	60%	0	-	-
Mohan 2020 [10]	48	CellSearch	77%	39	2	65%	30	11	86%
Igawa 2014 [46]	30	OBP-401	96%	8	-		22	-	
Wang 2017 [23]	42	Negative immunomagnetic enrichment and anti-CD45 and anti-panCK antibodies	86%	17	NA, median overall 5	N.A.	25	NA, median overall 5	NA
Messaritakis 2019 [49]	108	CellSearch	80%	37	NA, median overall 14	N.A.	71	NA, median overall 14	NA
Messaritakis 2019 [49]	108	IF DLL3+/ CD45-	74%	37	-	-	71	-	-
Messaritakis 2018 [57]	66	IF BCL2+/CD45-	73%	26	-	-	40	-	-
Messaritakis 2017 [36]	108		43-61%	37	-	-	71	-	-

		IF<br>TTF1+/CD56<br>+/CD45-							
Su 2019 [32]	48	CellSearch	NA	8	NA, Median Overall 167	NA	40	NA, Median Overall 167	NA

^ 3 patients with recurrent disease. * Blood samples of 6 mL.

Table S4. Studies included in meta-analysis of prognostic value of baseline CTCs assessed by CellSearch.

REF.<br>Country	N.<br>(ES/LS)	Baseline CTC median number, (Min-Max)	Study type	Value	CTC Cut-off	HR<br>(HR 0.025 - HR 0.975)	Method	p	Quality (Newcastle-Ottawa Scale)
Mohan 2019 [10]<br>UK	55<br>(29/26)	Total: unknown,<br>LS (2.0-116),<br>ES (11.0-15,352)	Retro	OS	**>7**	**2.57 (1.31 - 5.06)**	UVA	0.00616	7/9
Tay 2019 [55]<br>UK	75<br>(0/75)	1 (0–3750)	Prosp	OS<br>PFS<br>OS<br>PFS<br>OS<br>PFS	**>15**<br>>15<br>>=2<br>>=2<br>>50<br>>50	**6.19 (3.08 - 12.42)**<br>6.03 (3.00 - 12.08)<br>2.15 (1.25 - 3.70)<br>1.85 (1.10 - 3.12)<br>3.42 (1.52 - 7.67)<br>3.589 (1.57 - 8.18)	MVA<br>MVA<br>MVA<br>MVA<br>MVA<br>MVA	<0.001<br><0.001<br>0.006<br>0.021<br>0.003<br>0.002	6/9
Fu 2018 [56]<br>China	112<br>(0/112)	19.5 (2–247.5)	Retro	OS<br>PFS	**>19.5**<br>>19.5	**4.73 (1.05-13.9)**<br>2.37 (1.05-6.33)	MVA<br>MVA	0.01<br><0.01	6/9
Messaritakis 2017 * [36]<br>Greece	83<br>(54/29)	Total: 14 (0–10.000)<br>ES: nr<br>LS: nr	Prosp	OS<br>PFS	**>5**<br>>5	**3.4 (1.8-6.3)**<br>1.9 (0.9 -3.9)	UVA<br>MVA	<0.001<br>0.048	6/9
Salgia 2017 [42]<br>USA	61<br>(61/0)	51.0 (0-21428)	Phase II	OS<br>PFS	**>6**<br><6	**2.43 (1.14-5.16)^**<br>0.472 (0.242 -0.919)	MVA<br>MVA	nr<br>nr	6/9
Belani 2016 [38]<br>USA	120<br>(nr/nr)	nr	Phase II	OS<br>PFS	**>100**<br>>100	**1.74 (1.17-2.61)**<br>1.69 (1.13-2.53)	MVA<br>MVA	0.006<br>0.01	5/9
Normanno 2014 [60]<br>Italy	60<br>(60/0)	47 (0-24281)	Prosp	OS<br>OS<br>OS<br>OS	**>2**<br><282<br><50<br><8	1.59 (0.85-2.96)**^<br>0.5 (0.26-0-95)<br>0.63 (0.34 -1.18)<br>0.37 (0.16-0.87)	MVA<br>MVA<br>MVA<br>MVA	nr<br>nr<br>nr<br>nr	6/9
Hiltermann 2012 [18]<br>Netherlands	59<br>(38/21)	Total: 59 (16; 0–14040)<br>LS: (6.0–220),<br>ES: (63.0–14 040)	Prosp	OS<br>PFS	**>2**<br>>2	**2.3 (0.8 – 6.3)**<br>3 (1.4-6.6)	MVA<br>UVA	0.11<br>nr	7/9
Naito 2012 [17]<br>Japan	51<br>(24/27)	Total: 4 (0–5648),<br>ES 9.5 (0–5648),<br>LS 1 (0–58)	Prosp	OS<br>OS<br>OS	**>8**<br>>2<br>>5	3.5 (1.45-8.6)***<br>0.67 (0.25-1.87)<br>1.59 (0.61-4,29)	MVA<br>MVA<br>MVA	0.0014<br>nr<br>nr	6/9
Hou 2012 [16]<br>UK	97<br>(66/31)	Total: 24 (0-44.896)<br>ES: nr<br>LS: 1 (0-91)	Prosp	OS<br>PFS	**>50**<br>>50	**2.45 (1.39-4.3)**<br>2.01 (1.17-3.46)	MVA<br>MVA	0.002<br>0.011	6/9

Hou 2009 [40] UK	88 (53/35)	Total: 28 (0–44.896), ES: 237 (1–44896) LS: 2 (0–91)	Prosp	OS	**>300 vs <2**	**1.1 (1.02-1.18)^^**	UVA	0.015	6/9

When HR was not studied for a certain cut-off we showed Kaplan Meyer curves in months. *Partially the same population of Messaritakis 2018 [57] which was not included in this meta-analysis because of a smaller sample size. ** A cut-off of 2 CTCs had the highest C index (>0.728 vs 0.712 of 282 CTC cut off). *** Multiple cut-offs studied, we reported the different results obtained with different cut-offs, and we chose those with the better operating characteristics throughout ROC analysis for the meta-analysis (*bold*). ^: HR were inverted because, in these cases, the control group presented a higher number of CTCs than the cut-off. ^^: not significant in multivariate analysis, but HR not reported. CTCs: circulating tumor cells; ES: extensive stage; HR: hazard ratio; LS: limited stage; MVA: multivariate analysis; OS: overall survival; PFS: progression free survival; Prosp: prospective; Retro: retrospective; UVA: univariate analysis.
